# Plant Stress Detection via Molecular Communication: Modeling BVOC-Based Inter-Plant Signaling for Agricultural Monitoring

**DOI:** 10.3390/plants14182874

**Published:** 2025-09-16

**Authors:** Yusheng Sun, Pengfei Zhang, Pengfei Lu

**Affiliations:** College of Information Science and Technology, Shihezi University, Shihezi 832003, China; 20232108025@stu.shzu.edu.cn (Y.S.); 20232108002@stu.shzu.edu.cn (P.Z.)

**Keywords:** plant communication, stress detection, molecular communication, abiotic and biotic stress, testbed

## Abstract

In the plant kingdom, stress can significantly impact physiological and metabolic processes, leading to growth inhibition, developmental abnormalities, and even mortality. Current detection methods primarily focus on changes in gene expression or observable disease symptoms. However, these approaches are often resource-intensive, costly, and procedurally complex. To overcome these challenges, this study introduces an innovative molecular communication framework for plant stress monitoring. In this framework, plants that release biogenic volatile organic compounds serve as transmitters, receiving plants act as receivers, and the air serves as the propagation channel. The primary objective is to develop a real-time stress detection method by modulating stress types into distinct profiles of biogenic volatile organic compounds. These profiles are transmitted as chemical signals and are demodulated at the receiver. We analyzed the effects of distance, wind speed, and other factors on compound dispersion in the channel, validating the system through simulations and a molecular communication testbed. This research provides an innovative technical approach for real-time plant stress monitoring while establishing a theoretical foundation for enhancing crop management efficiency and advancing precision agriculture.

## 1. Introduction

Plants, as vital components of ecosystems, play an essential role in maintaining ecological balance and alleviating global climate change. Research in relation to plant physiology primarily focuses on how essential metabolic processes (photosynthesis, respiration, and nutrient assimilation) sustain life and coordinate growth in response to dynamic environmental conditions [1,2]. However, these processes do not occur in isolation. In natural settings, environmental fluctuations (variations in light intensity, temperature variations, and water availability fluctuations) are the norm for plant survival. When these fluctuations exceed a plant’s homeostatic capacity, they induce stress. Although stress may inhibit short-term growth, it also drives plants to evolve complex molecular sensing networks and systemic defense mechanisms [3]. Plant stress can be classified into biotic and abiotic types based on its origin. Biotic stress results from damage caused by other organisms, including infections by pathogens (bacteria, fungi, and viruses), infestations by pests, and grazing by animals. In contrast, abiotic stress is caused by extreme changes in physical or chemical environmental factors, such as drought, high temperatures, and soil salinization [4,5].

**Figure 1:**
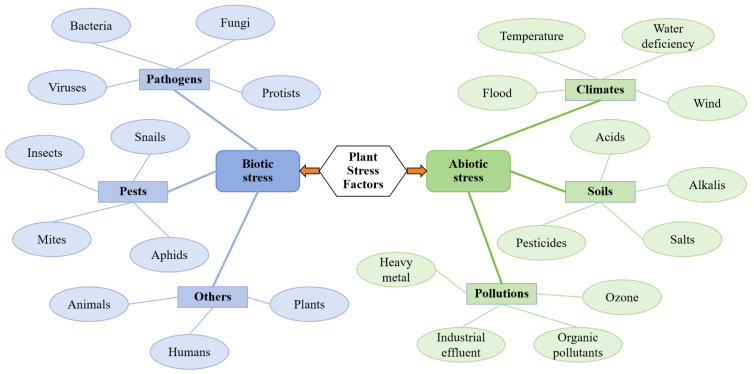
Biotic and abiotic stress factors in plants (adapted from [9]).

According to statistics from the Food and Agriculture Organization (FAO), environmental stresses account for approximately 30% of annual global crop yield losses, with drought and pest infestations/diseases contributing 29% and 40% for these losses, respectively [6]. This alarming scenario underscores the urgent need to investigate the mechanisms underlying plant stress responses. A comprehensive understanding of both the molecular regulatory networks and physiological adaptation mechanisms involved in plant responses to biotic and abiotic stresses will not only lay a theoretical foundation for developing environmentally friendly plant protection strategies and breeding stress-tolerant crops but will also support efforts to enhance crop resilience under climate change conditions [7,8,9]. Thus, innovative research on systematic stress detection technologies emerges as a fundamental prerequisite for advancing these critical research areas (Figure 1).

Current research on plant stress detection focuses on three main approaches—electrochemical methods, hyperspectral imaging, and deep learning techniques. Among the electrochemical methods, three specific techniques have been developed, namely direct electrochemistry, immunoelectrochemistry, and photoelectrochemistry. However, these methods suffer from low adaptability and insufficient sensitivity when analyzing complex biological samples [10]. Hyperspectral imaging has shown promise in distinguishing biotic and abiotic stresses in *maize*, but its effectiveness is compromised by environmental interference, which remains insufficiently addressed [11]. Deep learning approaches, particularly the Faster R-CNN algorithm, have achieved 76.07% accuracy in detecting canopy stresses with similar symptoms in *tea* plants. While these models demonstrate reasonable precision, they are highly dependent on large datasets and exhibit poor cross-environment generalization capabilities [12]. Chlorophyll fluorescence imaging enables early stress detection through excitation–emission signal analysis [13]. Near-infrared fluorescence combined with surface-enhanced Raman scattering (SERS) provides improved diagnostic specificity, but is hampered by high equipment costs and cumbersome operational procedures [14]. A novel embedded sensor demonstrated laboratory-grade sensitivity in *grapevine* dehydration experiments, though its practical field applicability requires further validation relating to its long-term stability and durability performance [15].

In summary, existing plant stress detection methods face several key challenges—high costs, complex sample processing procedures, limited environmental adaptability, and susceptibility to interference. More importantly, there is a critical knowledge gap in the systematic quantitative analysis of stress signal transmission pathways and detection mechanisms across plant populations—a gap that hinders our understanding of plant communication networks and ecological adaptation strategies under stress conditions. To address these limitations, this study proposes a molecular communication (MC) framework [16,17]. As an emerging interdisciplinary field that integrates bio-inspired principles with communication engineering, MC enables the quantitative modeling of propagation dynamics for biogenic volatile organic compounds (BVOCs) under diverse stress conditions [18]. Building upon this foundation, we propose a novel analytical framework for plant stress detection. Within this framework, we have developed mathematical models that describe the complete lifecycle or BVOCs—including generation, release, atmospheric propagation, inter-plant absorption, and detection—while quantifying the impact of environmental factors such as inter-plant distance and ambient wind speed on the efficiency and accuracy of stress signal transmission.

The primary contributions of this paper are as follows:

1. We established an MC model for plant stress detection that provides a comprehensive theoretical framework characterizing BVOC dynamics, including release mechanisms, transmission pathways, and absorption processes. Furthermore, we have derived mathematical formulations for the demodulation processes and developed quantitative performance metrics, notably multi-molecular detection error rates (DERs), to evaluate system performance.
2. We simulated the MC model to quantify how transmission distance, wind speed, and BVOC release quantity impact its performance.
3. We developed an MC testbed that leverages two distinct pigment dyes as proxies for authentic BVOCs to experimentally detect and differentiate between pest stress and heat stress, thereby providing empirical validation of the proposed model’s accuracy and effectiveness.

## 2. Materials and Methods

### 2.1. System Description

**Figure 2:**
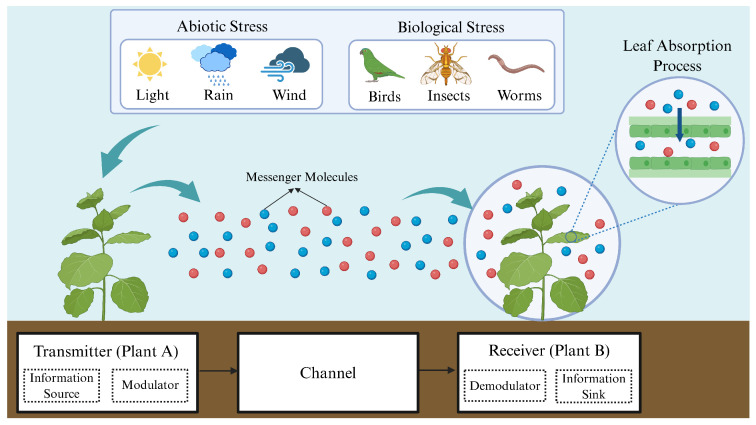
An MC framework between plants. The left-side plant (A), acting as the sender, transmits stress signals to the receiver plant (B) on the right by releasing BVOCs upon stress exposure. In this process, plant A modulates the source stress into corresponding molecular signals, while plant B demodulates the received signals to identify the specific stress type. Additionally, the figure depicts BVOC penetration through mesophyll cell layers into the leaf interior, enabling intracellular signal propagation and processing. Figure created with https://BioRender.com.

To achieve the quantitative transmission and analysis of plant stress signals, this study breaks through the limitation of traditional plant signal research, which only focuses on a single link, and innovatively constructs a Single-Input Single-Output (SISO) MC system. This novel framework holistically models the entire signaling process, from stress perception and molecular emission to signal propagation and reception, thereby enabling a systematic investigation of plant response mechanisms under diverse stress conditions. The core structure of the system is shown in Figure 2. Plant A (the transmitter) modulates and releases BVOCs; the central channel is where BVOCs act as signaling molecules; and Plant B (the receiver) perceives and demodulates the BVOCs to identify stress types and activate appropriate defensive responses [19,20]. Furthermore, to ensure model tractability within experimental constraints, we implement three key assumptions. First, the analysis considers only biotic and abiotic stresses that are known to induce volatile organic compound emission. Second, each stress type triggers the emission of a single characteristic compound, with no cross-interference between molecular species. Third, the channel maintains stable environmental conditions and assumes negligible molecular degradation throughout signal propagation.

### 2.2. Transmitter

In this unique plant-to-plant communication system, stress information originates from various stimuli experienced by plants [21]. Transmitter plants release stress-specific BVOCs to communicate physiological status to neighboring receivers—a process aligned with the MC principle of multi-molecular type modulation [17], where distinct molecular species modulate different messages. In plant stress signaling, this modulation mechanism capitalizes on the species specificity of BVOCs, whereby distinct stress types induce the emission of unique molecular profiles. This process effectively translates stress identity into a discriminative molecular identity, thereby facilitating reliable inter-plant communication [22].

Receiver plants preset a minimum detection threshold for each stress-specific BVOC, a trait rooted in their physiological adaptation that ensures targeted defensive responses are activated only upon reaching biologically significant signal levels. This process follows a “single-molecule threshold activation with multi-molecule mutual exclusion” principle to guarantee unique stress identification. Specifically, we first define the set of stress types experienced by transmitter plants as $S=\{s_{1},s_{2},\ldots ,s_{m},s_{m+1},s_{m+2},\ldots ,s_{n}\}$, where biological stressors are denoted as $\left\{s_{1},s_{2},\ldots ,s_{m}\right\}$ and abiotic stressors are denoted as $\left\{s_{m+1},s_{m+2},\ldots ,s_{n}\right\}$. Through multi-molecular-type modulation, we map each stress type to a unique BVOC, forming the BVOC set $B=\{b_{1},b_{2},\ldots ,b_{n}\}$, where there is a one-to-one correspondence between $s_{i}$ and $b_{i}$. The stress demodulation process of the receiver plant can be described by the following mathematical formula:

(1) $$S\left[k\right]=\left\{\begin{array}{cc} s_{1}, & M_{\mathrm{abs}}^{b_{1}}\left[k\right]\geq \theta_{b_{1}},M_{\mathrm{abs}}^{b_{2}}\left[k\right]<\theta_{b_{2}},\ldots ,M_{\mathrm{abs}}^{b_{n}}\left[k\right]<\theta_{b_{n}},\\ s_{2}, & M_{\mathrm{abs}}^{b_{2}}\left[k\right]\geq \theta_{b_{2}},M_{\mathrm{abs}}^{b_{1}}\left[k\right]<\theta_{b_{1}},\ldots ,M_{\mathrm{abs}}^{b_{n}}\left[k\right]<\theta_{b_{n}},\\ \vdots & \\ s_{n}, & M_{\mathrm{abs}}^{b_{n}}\left[k\right]\geq \theta_{b_{n}},M_{\mathrm{abs}}^{b_{1}}\left[k\right]<\theta_{b_{1}},\ldots ,M_{\mathrm{abs}}^{b_{n-1}}\left[k\right]<\theta_{b_{n-1}},\\ \mathrm{error}, \end{array}\right.$$

where S[k] denotes the stress experienced by the transmitter plant during the k symbol interval, while $M_{\mathrm{abs}}^{b_{1}}\left[k\right]$ represents the quantity of $b_{1}$ absorbed by the receiver plant’s leaves during the k symbol interval reception time slot under $s_{1}$ stimulation. $\theta_{b_{1}}$ signifies the detection threshold of the receiver plant for $b_{1}$. The definitions of other symbols ($M_{\mathrm{abs}}^{b_{2}}\left[k\right]$) follow an analogous convention. Additionally, an error occurs when either no absorbed molecules or all absorbed molecules meet or exceed their respective detection thresholds, resulting in erroneous stress type identification by the receiver plant.

We have clarified the stress identification logic of receiver plants based on BVOC detection through Equation (1). However, the release of BVOCs by transmitter plants is predicated on the stress-induced BVOC biosynthesis and emission process—this process relies on complex biological mechanisms within plants such as gene expression regulation and signal pathway transduction, directly determining the production rate and emission amount of BVOCs, as well as constituting the core of the “signal generation” link in inter-plant communication [23]. To model this process, we introduce two key simplifying assumptions—the regulatory effect is additive and captured through weighted inputs and no autoregulation occurs in this model. Based on these assumptions, we now model the BVOC production process in transmitter plants to quantitatively capture the full pathway from stress stimulation to BVOC signal emission.Therefore, the rate of BVOC synthesis can be expressed as follows:

(2) $$\xi \left(t\right)=\frac{dG_{x}}{dt}=\frac{\eta_{x1}}{1+\exp\left(-a_{x0}-\sum_{y\in E}a_{xy}G_{y}\right)}-\eta_{x2}G_{x},$$

where $G_{x}$ and $G_{y}$ denote the expression levels of gene *x* and gene *y*, E represents the set of potential regulatory influencers for gene *x*, and $a_{x0}$ corresponds to the basal expression rate of gene *x* in the absence of regulatory factors. For each potential regulator *y*, $a_{xy}$ quantifies the regulatory effect of gene *y* on gene *x* (with the assumption of no autoregulation in this model). The maximum expression rate and decay rate of gene *x* are denoted by $\eta_{x1}$ and $\eta_{x2}$, respectively.

**Figure 3:**
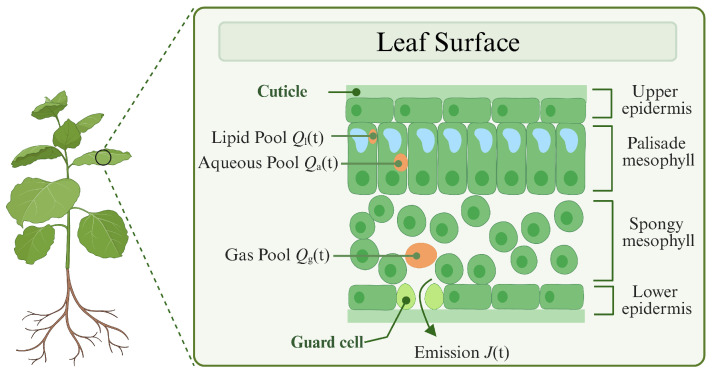
The internal structure of leaves and the mechanisms of BVOC release. Figure created with https://BioRender.com.

Upon production at a rate of ξ(t), BVOCs are initially transported across cellular membranes into distinct intracellular compartments, where they undergo selective partitioning into lipid-phase or aqueous-phase storage pools [24]. As illustrated in Figure 3, these volatile organic compounds then progressively diffuse through passive transport into the gas-phase reservoir within intercellular spaces, culminating in their regulated release into the atmosphere through stomatal regulatory mechanisms [25]. The trans-compartmental transport dynamics can be represented as follows:

(3) $$\frac{dQ_{a}\left(t\right)}{dt}=\eta \xi \left(t\right)-u_{a}Q_{a}\left(t\right),$$

(4) $$\frac{dQ_{l}\left(t\right)}{dt}=\left(1-\eta \right)\xi \left(t\right)-u_{l}Q_{l}\left(t\right),$$

(5) $$\frac{dQ_{g}\left(t\right)}{dt}=u_{a}Q_{a}\left(t\right)+u_{l}Q_{l}\left(t\right)-u_{g}Q_{g}g\left(t\right).$$

The partitioning of BVOCs between aqueous and lipid phases is quantitatively described by the distribution coefficient η, where $Q_{a}\left(t\right)$ and $Q_{l}\left(t\right)$ denote the transient storage pools in the aqueous and lipid phases, respectively. $u_{a}$ denotes the rate of release from the aqueous storage pool, whereas $u_{l}$ denotes the rate of release from the lipid storage pool. Subsequently, BVOCs enter the gaseous storage pool $Q_{g}\left(t\right)$, with the emission rate $u_{g}$ into the ambient atmosphere being determined by the total gas-phase conductance across the cell wall interface [26]. Thus, the release rate of BVOCs can be expressed as follows:

(6) $$J\left(t\right)=u_{g}Q_{g}\left(t\right).$$

Subsequently, by convolving J(t) with the channel impulse response, the cumulative release amount from the transmitter plant for $b_{i}$ up to the given time t can be obtained as follows:

(7) $$M_{b_{i}}\left(t\right)=\int_{0}^{t}J\left(\delta \right)f_{b_{i}}\left(t-\delta \right)\;d\delta .$$

where $b_{i}$ represents a molecular species from B; $f_{b_{i}}$ denotes the channel impulse response of $b_{i}$.

### 2.3. Channel

**Figure 4:**
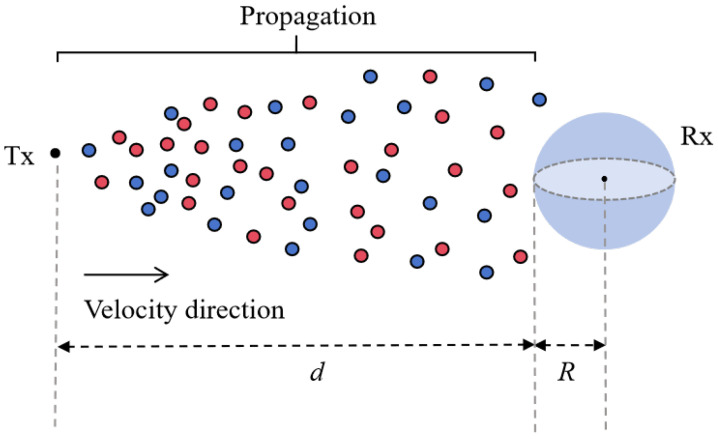
The propagation process of BVOCs. Tx represents the transmitter, Rx denotes the receiver, the red and blue circles represent different types of BVOCs released by plants, which are transported under the influence of horizontal wind speed.

In this section, we analyze the propagation process of BVOCs and establish a corresponding channel model. To simplify the analysis, the emitting plants will be treated as point sources, while the receiver plants will be modeled as a perfectly absorbing sphere with a radius *R*. The distance between the transmitter and receiver is denoted as *d*, and a variable wind speed will be included in the channel to simulate natural environmental conditions, as illustrated in Figure 4.

After being released, BVOCs undergo both diffusive motion caused by random collisions with surrounding gas molecules and convective transport driven by airflow. The successful detection of these molecules by receiver plants depends on the molecules’ spatial trajectories and the combined effects of external environmental conditions. To accurately characterize the propagation dynamics of BVOCs in the propagation channel, we utilize the convection–diffusion equation for mathematical modeling, which is expressed as follows:

(8) $$\frac{\partial f_{b_{i}}\left(d,t\right)}{\partial t}+\nabla vf_{b_{i}}\left(d,t\right)=D_{b_{i}}\nabla^{2}f_{b_{i}}\left(d,t\right),$$

where $b_{i}$ represents a molecular species from B; $f_{b_{i}}\left(d,t\right)$ denotes the probability density function of $b_{i}$ within the channel. Different BVOCs exhibit distinct diffusion characteristics, with $D_{b_{i}}$ representing the diffusion coefficient of $b_{i}$. *v* indicates wind speed. The gradient operator ∇ and the Laplace operator $\nabla^{2}$ describe the spatial variation and diffusion effects, respectively.

Under the specified initial condition (all molecules are concentrated at the emission point with instantaneous release at t = 0) and boundary conditions (complete absorption upon reaching the receiver surface and asymptotic decay of probability density at infinity), the analytical solution $f_{b_{i}}\left(d,t\right)$ to Equation (8) is obtained as follows [16]:

(9) $$f_{b_{i}}\left(d,t\right)=\frac{Rd}{\left(R+d\right)\sqrt{4\pi D_{b_{i}}t^{3}}}\exp\left(-\frac{{\left(d-vt\right)}^{2}}{4D_{b_{i}}t}\right).$$

To systematically analyze the optimal detection timing at the receiver under different parameter combinations, we need to determine the extremum points of $f_{b_{i}}\left(d,t\right)$, as follows:

(10) $$\frac{\partial f_{b_{i}}\left(d,t\right)}{\partial t}=0.$$

Solving Equation (10) yields the following peak time:

(11) $$t_{\mathrm{peak}}=\frac{-6D_{b_{i}}+\sqrt{36D_{b_{i}}^{2}+4v^{2}d^{2}}}{2v^{2}}.$$

Furthermore, by integrating Equation (9) over time, the cumulative reception probability for $b_{i}$ at the receiver plant up to a given time instant ω can be obtained as follows:

(12) $$F_{\mathrm{hit}}^{b_{i}}=\int_{0}^{\omega }f_{b_{i}}\left(d,t\right)\;dt.$$

Therefore, the total quantity of $b_{i}$ reaching the receiver during this period is denoted by $MF_{\mathrm{hit}}^{b_{i}}$. While plant leaves serve as the primary absorption sites for BVOCs, interspecific differences exist in leaf number, size, and morphology. Furthermore, the actual leaf volume does not entirely occupy the modeled absorption sphere. To account for this discrepancy, we introduce the volume ratio ϕ between the total leaf volume and the absorption sphere volume. Consequently, the concentration of $b_{i}$ on the receiver plant’s leaves can be expressed as follows:

(13) $$C_{\mathrm{leaf}}^{b_{i}}=\frac{3M_{b_{i}}F_{\mathrm{hit}}^{b_{i}}}{4\pi R^{3}\phi }.$$

By incorporating Equations (9) and (12), we obtain the following:

(14) $$C_{\mathrm{leaf}}^{b_{i}}=\frac{3M_{b_{i}}}{4\pi R^{2}\phi \left(R+d\right)}\int_{0}^{\omega }\frac{d}{\sqrt{4\pi D_{b_{i}}t^{3}}}\exp\left(-\frac{{\left(d-vt\right)}^{2}}{4D_{b_{i}}t}\right)dt.$$

### 2.4. Receiver

In the framework of plant communication, the primary function of the receiver is to detect and demodulate BVOCs transmitted through the channel. When BVOCs reach the receiver’s leaves, a fraction is absorbed and engages in downstream signal transduction and physiological responses, while the remaining portion is metabolized and subsequently re-emitted into the atmosphere [27]. Therefore, it is necessary to model this absorption process, which is mathematically expressed as follows:

(15) $$\frac{dC_{\mathrm{abs}}^{b_{i}}\left(t\right)}{dt}=\frac{C_{\mathrm{leaf}}^{b_{i}}A_{L}g}{m_{L}}-\frac{A_{L}\rho_{L}g}{K_{L}m_{L}}C_{\mathrm{abs}}^{b_{i}}\left(t\right),$$

where $C_{\mathrm{abs}}^{b_{i}}$ denotes the concentration of $b_{i}$ in the leaf tissue, $A_{L}$ represents the leaf area, *g* is the leaf conductance, $\rho_{L}$ indicates the leaf density, $m_{L}$ corresponds to the leaf mass, and $K_{L}$ is the leaf–air partition coefficient. Based on Equation (14), the value of $C_{\mathrm{leaf}}^{b_{i}}$ can be obtained, allowing for the determination of the steady-state solution for $C_{\mathrm{abs}}^{b_{i}}$, as follows:

(16) $$C_{\mathrm{abs}}^{b_{i}}\left(t\right)=\frac{K_{L}C_{\mathrm{leaf}}^{b_{i}}}{\rho_{L}}\left(1-\exp\left(-\frac{A_{L}\rho_{L}g}{K_{L}m_{L}}t\right)\right).$$

Based on these results, we can further derive the cumulative absorption quantity of $b_{i}$ in the receiver plant leaves over a given absorption period τ, as follows:

(17) $$M_{\mathrm{abs}}^{b_{i}}=\frac{3M_{b_{i}}F_{\mathrm{hit}}^{b_{i}}m_{L}K_{L}}{4\pi R^{3}\phi \rho_{L}}\int_{0}^{\tau }\left(1-\exp\left(-\frac{A_{L}\rho_{L}g}{K_{L}m_{L}}t\right)\right)dt.$$

Subsequently, we can derive the DER for $b_{i}$ based on the detection criteria established in Equation (1). Under long-distance transmission conditions, the propagation delays of BVOCs are non-negligible, and this factor necessitates the optimization of strategies for selecting detection time slots. In this study, we adopt the peak concentration time $t_{\mathrm{peak}}$ as the center, with a duration of $T_{s}$ or the detection time slot, extending $\pm \frac{1}{2}T_{s}$. Thus, the detection interval for the kth interval is given by $\left[\left(k-1\right)T_{s}+t_{\mathrm{peak}}-\frac{1}{2}T_{s},\;\left(k-1\right)T_{s}+t_{\mathrm{peak}}+\frac{1}{2}T_{s}\right]$. Accordingly, based on Equation (12), the cumulative reception rate for $b_{i}$ at the receiver during the kth interval can be expressed as follows:

(18) $$q_{k}^{b_{i}}=F_{\mathrm{hit}}^{b_{i}}\left(\left(k-1\right)T_{s}+t_{\mathrm{peak}}+\frac{1}{2}T_{s}\right)-F_{\mathrm{hit}}^{b_{i}}\left(\left(k-1\right)T_{s}+t_{\mathrm{peak}}-\frac{1}{2}T_{s}\right).$$

Therefore, the total quantity of $b_{i}$ reaching the receiver plant during the kth intervals can be expressed as follows:

(19) $$M_{k}^{b_{i}}=M_{c,k}^{b_{i}}+M_{ISI,k}^{b_{i}}+M_{n,k}^{b_{i}},$$

where $M_{c,k}^{b_{i}}$ is the quantity of received $b_{i}$ during the kth interval which $b_{i}$ transmitted at the beginning of kth interval, $M_{ISI,k}^{b_{i}}$ is the quantity of $b_{i}$ received during the kth interval but transmitted from the previous interval and defined as ISI, and $M_{n,k}^{b_{i}}$ represents the ambient noise caused by $b_{i}$ emissions from neighboring plants. $M_{c,k}^{b_{i}}$ can be modeled as a binomial distribution $M_{c,k}^{b_{i}}∼B\left(M_{b_{i}},q_{1}^{b_{i}}\right)$. When $M_{c,k}^{b_{i}}$ is sufficiently large, this binomial distribution can be approximated by a Faussian distribution, which is expressed as follows:

(20) $$M_{c,k}^{b_{i}}∼N\left(M_{b_{i}}q_{1}^{b_{i}},M_{b_{i}}q_{1}^{b_{i}}\left(1-q_{1}^{b_{i}}\right)\right),$$

where $q_{1}^{b_{i}}$ is the probability of $b_{i}$ transmitted at the beginning of the kth interval and observed by the receiver during the kth interval. Similarly, $M_{ISI,k}^{b_{i}}$ can also be modeled as a Gaussian distribution, as follows:

(21) $$M_{ISI,k}^{b_{i}}=\sum_{\lambda =1}^{k-1}N\left(p_{i}M_{b_{i}}q_{k+1-\lambda }^{b_{i}},p_{i}M_{b_{i}}q_{k+1-\lambda }^{b_{i}}\left(1-q_{k+1-\lambda }^{b_{i}}\right)\right),$$

where $p_{i}$ denotes the probability of $b_{i}$ transmission for each time slot; $q_{k+1-\lambda }^{b_{i}}$ denotes the probability that $b_{i}$ transmitted at the λth time slot is successfully received at the kth interval. Similarly, $M_{n,k}^{b_{i}}$ can also be modeled as a normally distributed random variable representing the system noise, as follows:

(22) $$M_{n,k}^{b_{i}}∼N\left(\mu_{n}^{b_{i}},{\left(\sigma_{n}^{b_{i}}\right)}^{2}\right),$$

where $\mu_{n}^{b_{i}}$ is the average received noise quantity of $b_{i}$; $\sigma_{n}^{b_{i}}$ represents the standard deviation, dependent on $\mu_{n}^{b_{i}}$. Based on the cumulative characteristics of the distribution, $M_{k}^{b_{i}}$ can be expressed as follows:

(23) $$M_{k}^{b_{i}}∼N\left(\mu_{k}^{b_{i}},{\left(\sigma_{k}^{b_{i}}\right)}^{2}\right),$$

where

(24) $$\mu_{k}^{b_{i}}=M_{b_{i}}q_{1}^{b_{i}}+\sum_{\lambda =1}^{k-1}p_{i}M_{b_{i}}q_{k+1-\lambda }^{b_{i}}+\mu_{k}^{b_{i}},$$

(25) $${\left(\sigma_{k}^{b_{i}}\right)}^{2}=M_{b_{i}}q_{1}^{b_{i}}\left(1-q_{1}^{b_{i}}\right)+\sum_{\lambda =1}^{k-1}p_{i}M_{b_{i}}q_{k+1-\lambda }^{b_{i}}\left(1-q_{k+1-\lambda }^{b_{i}}\right)+{\left(\sigma_{n}^{b_{i}}\right)}^{2}.$$

Subsequently, according to Equation (17), the quantity of $b_{i}$ absorbed by the plant leaf at the receiver during the kth interval, which is denoted as follows:

(26) $$M_{k,\mathrm{abs}}^{b_{i}}=M_{k}^{b_{i}}\frac{3m_{L}K_{L}}{4\pi R^{3}\rho_{L}\phi }\int_{0}^{\tau }\left(1-\exp\left(-\frac{A_{L}\rho_{L}g}{K_{L}m_{L}}t\right)\right)dt.$$

The mean quantity of $b_{i}$ after absorption can be expressed as follows:

(27) $$\mu_{k,\mathrm{abs}}^{b_{i}}=\mu_{k}^{b_{i}}\frac{3m_{L}K_{L}}{4\pi R^{3}\rho_{L}\phi }\int_{0}^{\tau }\left(1-\exp\left(-\frac{A_{L}\rho_{L}g}{K_{L}m_{L}}t\right)\right)dt.$$

The variance can be expressed as follows:

(28) $${\left(\sigma_{k,\mathrm{abs}}^{b_{i}}\right)}^{2}={\left(\sigma_{k}^{b_{i}}\right)}^{2}{\left(\frac{3m_{L}K_{L}}{4\pi R^{3}\rho_{L}\phi }\int_{0}^{\tau }\left(1-\exp\left(-\frac{A_{L}\rho_{L}g}{K_{L}m_{L}}t\right)\right)dt\right)}^{2}.$$

Thus, we obtain the DER of $b_{i}$ released in the kth interval after being absorbed by the receiver plant, as follows:

(29) $$\begin{array}{cc} P(k=b_{i}) & =1-P\left(M_{k,\mathrm{abs}}^{b_{i}}\geq \theta_{b_{i}}\right)\times \prod_{j=1,j\neq i}^{n}P\left({\hat{M}}_{k,\mathrm{abs}}^{b_{j}}<\theta_{b_{j}}\right)\\ \; & =1-Q\left(\frac{\theta_{b_{i}}-\mu_{k,\mathrm{abs}}^{b_{i}}}{{\left(\sigma_{k,\mathrm{abs}}^{b_{i}}\right)}^{2}}\right)\times \prod_{j=1,j\neq i}^{n}Q\left(\frac{{\hat{\mu }}_{k,\mathrm{abs}}^{b_{j}}-\theta_{b_{j}}}{{\left({\hat{\sigma }}_{k,\mathrm{abs}}^{b_{j}}\right)}^{2}}\right), \end{array}$$

where Q(x) denotes the Q-function, which represents the right-tail probability of the standard normal distribution. It is defined as the integral of the standard normal probability density function from *x* to infinity. ${\hat{M}}_{k,\mathrm{abs}}^{b_{j}}$ represents the quantity of $b_{j}$ released in all previous time slots up to the kth slot that are absorbed in the current time slot. This can be similarly modeled by a normal distribution, ${\hat{M}}_{k,\mathrm{abs}}^{b_{j}}∼N\left({\hat{\mu }}_{k,\mathrm{abs}}^{b_{j}},{\left({\hat{\sigma }}_{k,\mathrm{abs}}^{b_{j}}\right)}^{2}\right)$, where ${\hat{\mu }}_{k,\mathrm{abs}}^{b_{j}}$ and ${\left({\hat{\sigma }}_{k,\mathrm{abs}}^{b_{j}}\right)}^{2}$ represent the estimated mean and variance, respectively, given as follows:

(30) $${\hat{\mu }}_{k,\mathrm{abs}}^{b_{j}}=\left(\sum_{\lambda =1}^{k-1}p_{j}M_{b_{i}}q_{k+1-\lambda }^{b_{j}}+\mu_{k}^{b_{j}}\right)\frac{3m_{L}K_{L}}{4\pi R^{3}\rho_{L}\phi }\int_{0}^{\tau }\left(1-\exp\left(-\frac{A_{L}\rho_{L}g}{K_{L}m_{L}}t\right)\right)dt,$$

(31) $$\;{\left({\hat{\sigma }}_{k,\mathrm{abs}}^{b_{j}}\right)}^{2}=\left(\sum_{\lambda =1}^{k-1}p_{j}M_{b_{i}}q_{k+1-\lambda }^{b_{j}}\left(1-q_{k+1-\lambda }^{b_{j}}\right)+{\left(\sigma_{n}^{b_{j}}\right)}^{2}\right){\left(\frac{3m_{L}K_{L}}{4\pi R^{3}\rho_{L}\phi }\int_{0}^{\tau }\left(1-\exp\left(-\frac{A_{L}\rho_{L}g}{K_{L}m_{L}}t\right)\right)dt\right)}^{2}.$$

## 3. Results

In this section, to investigate how model parameters influence stimulus recognition, we conducted numerical simulations in MATLAB (R2022b), systematically analyzing the effects of distance, wind speed, and related parameters on absorption and DER. The settings for distance and wind speed were derived from the proposed BVOC atmospheric propagation model, enabling us to examine how they affect the probability that plants receive BVOCs. In addition, we quantified the number of BVOC molecules absorbed by plants to simulate the proposed leaf absorption model and the cumulative reception probability. DER was then computed based on the absorption, which allowed us to validate how various factors impact the correct recognition of stimuli. Finally, we built an experimental test platform to provide a preliminary validation for recognizing two types of stimuli. In future work, we will further refine the platform and strengthen the underlying theoretical framework. To maintain model fidelity, Table 1 presents the critical system parameters utilized in our analysis. These parameters comprise the leaf quality $m_{L}$ and the leaf area $A_{L}$, sourced from [28]; the leaf electric conductance *g* and the leaf density $\rho_{L}$ from [29]; and the air diffusion coefficient $K_{L}$ from [30].

### 3.1. Impact of Factors on Leaf Absorption

The absorption of BVOCs by plant leaves is influenced by multiple factors, which include not only the intrinsic properties of BVOCs (diffusion coefficients and emission quantities) but also key environmental conditions (wind speed). This section quantitatively examines these determinants to elucidate the underlying mechanisms by which plants accurately recognize and decode stress signals from neighboring plants under varying environmental conditions.

#### 3.1.1. Distance Analysis

**Figure 5:**
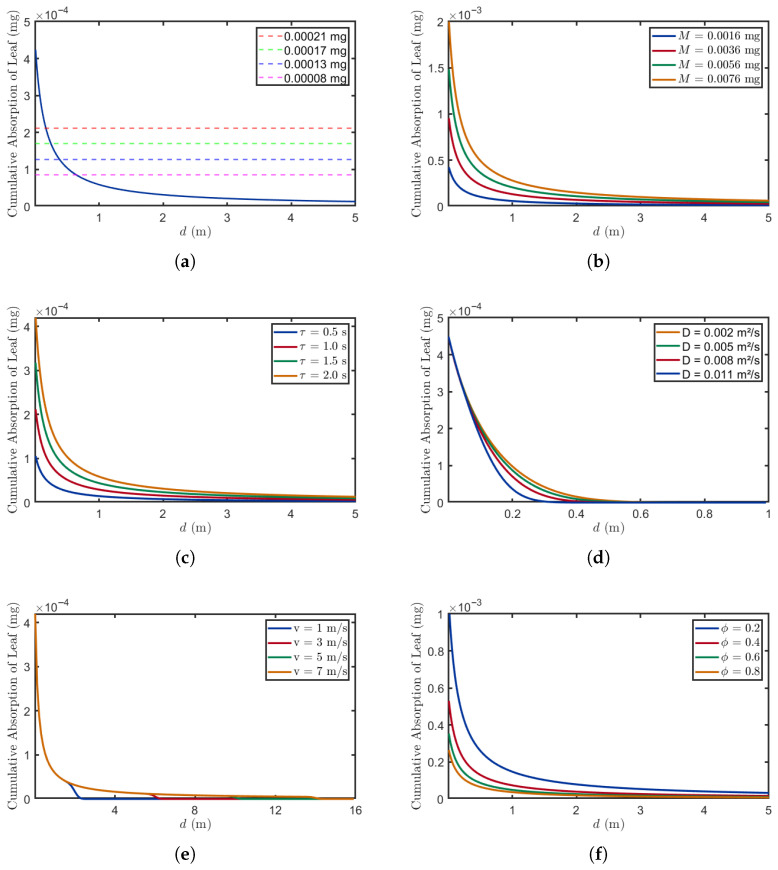
The relationship between distance and absorption under different parameters. (**a**) M=0.0016mg, v=3m/s, $D=0.008\;m^{2}/s$, ω=2s, τ=2s, ϕ=0.5. (**b**) v=3m/s, $D=0.008\;m^{2}/s$, ω=2s, τ=2s, ϕ=0.5. (**c**) M=0.0016mg, v=3m/s, $D=0.008\;m^{2}/s$, ω=2s, ϕ=0.5. (**d**) M=0.0016mg, v=0.5m/s, ω=2s, τ=2s, ϕ=0.5. (**e**) M=0.0016mg, $D=0.008\;m^{2}/s$, ω=2s, τ=2s, ϕ=0.5. (**f**) M=0.0016mg, v=3m/s, $D=0.008\;m^{2}/s$, ω=2s, τ=2s.

In this section, we analyze the distance-dependent absorption characteristics of BVOCs, as show in Figure 5. The figure illustrates how BVOC absorption varies with transmission distance *d* across different experimental conditions, while explicitly accounting for threshold effects, diffusion coefficients, leaf volume percentages, and other influencing factors. Our analysis demonstrates a clear inverse relationship between transmission distance and absorption efficiency, whereby as the propagation distance increases, the diffusion time of BVOCs through the air is prolonged. This extended diffusion reduces molecular concentration at the receiver, thereby decreasing the total absorption.

Figure 5a demonstrates how the threshold affects the successful detection of stress signals by receiver plants at different distances, where the blue solid line represents the absorption of BVOCs at various distances. The threshold represents the minimum BVOC absorption quantity required for accurate stress detection and subsequent physiological response activation. At higher thresholds (0.00021 mg), plants can only detect stress signals within close distances (0 m to 0.3 m). As the distance increases, BVOC absorption decreases due to atmospheric resistance and molecular diffusion—a phenomenon that necessitates lower thresholds (0.00008 mg) to maintain detection efficacy at extended distances.

Figure 5b demonstrates the relationship between different release amounts and the absorption of BVOCs. In this study, various initial release values were tested to investigate their impact on absorption rates [31]. The results indicate that with higher release amounts (0.0076 mg), plants can maintain relatively high absorption levels over longer distances (5 m), compared to those observed with lower release quantities (0.0016 mg). This suggests that elevated emissions not only extend the effective transmission range of stress signals but also enhance signal stability during atmospheric dispersion.

Figure 5c demonstrates the changes in BVOC absorption by leaves across different absorption durations. This process is directly linked to the opening and closing dynamics of leaf stomata. At the same distance, longer absorption durations (2 s) result in a higher absorption of BVOCs compared to shorter absorption durations (0.5 s); this is because prolonged stomatal opening enhances BVOC uptake, thereby improving the detection accuracy of stress signals emitted by source plants. However, at greater distances (5 m), higher BVOC losses during atmospheric dispersion reduce the quantity reaching the receiver, weakening the influence of stomatal opening time on absorption efficiency.

Figure 5d demonstrates the relationship between different diffusion coefficients and absorption amounts. The experimental data were obtained from [32]. At the same distance (0.2 m), absorption gradually decreases as the diffusion coefficient *D* increases; this phenomenon can be attributed to the enhanced diffusion of BVOCs in directions other than the downwind direction. Furthermore, at greater distances (1 m), the variation in absorption amounts across different diffusion coefficients becomes negligible, suggesting that beyond a certain distance, environmental factors exert a more dominant influence over diffusion effects.

Figure 5e illustrates the relationship between different wind speeds and the absorption of BVOCs. The wind speed range selected for this study corresponds to Beaufort Wind Scale categories 1 through 4. As the wind speed increases, the propagation distance of BVOCs significantly expands. At lower wind speeds (1m/s), leaves can absorb a substantial amount of BVOCs at close distances (0.5 m), but absorption declines markedly beyond 2 m. This reduction occurs because molecular diffusion dominates under low wind speeds, resulting in inefficient absorption at greater distances. In contrast, at higher wind speeds (7m/s), convective processes become predominant, significantly enhancing the transmission distance of BVOCs. Additionally, the abrupt decreases observed at low wind speeds can be attributed to numerical instability in the computation of the hit probability integral. Under these conditions, the integral becomes sensitive to discretization parameters, leading to sudden changes in the simulated absorption. It is important to note that these drops are computational artifacts rather than indicative of real biological processes. In actual plant environments, we would expect a smoother concentration decay with distance, devoid of such sudden fluctuations.

Figure 5f demonstrates the relationship between different volume ratios and the absorption of BVOCs. The results indicate that for a given distance, a higher volume ratio (ϕ= 0.8) results in less BVOC absorption by leaves compared to a lower volume ratio (ϕ= 0.2). This occurs because an increase in the volume ratio reduces the concentration of BVOCs on the leaf surfaces of receiver plants, thereby impairing their BVOC absorption capacity.

#### 3.1.2. Propagation Time Analysis

**Figure 6:**
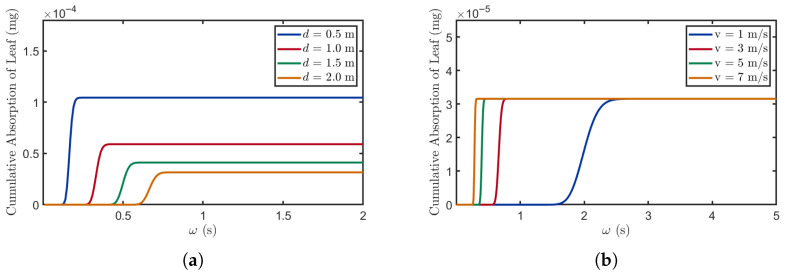
The relationship between propagation time and absorption quantity. (**a**) M=0.0016mg, v=3m/s, $D=0.008\;m^{2}/s$, τ=2s, ϕ=0.5. (**b**) M=0.0016mg, d=2m, $D=0.008\;m^{2}/s$, τ=2s, ϕ=0.5.

This section primarily explores the relationship between propagation time and leaf absorption. Figure 6 depicts the trends in leaf BVOC absorption under varying distance and wind speed conditions. As propagation time increases, the cumulative absorption of the leaves gradually increases.

Figure 6a shows that as distance decreases, the time at which leaves begin absorbing BVOCs occurs earlier, and the stable value of the absorption curve increases. This occurs because at shorter distances (0.5 m), the signaling molecules can reach the receiver plants more quickly. Additionally, due to the reduced loss of BVOCs during their propagation at close distances, the saturation absorption level of leaves is maximized. By shortening the distance between the sender and receiver plants, losses during the propagation process can be minimized, thereby strengthening the receiver plants’ ability to detect stress signals and providing them with an advantage in survival competition.

In Figure 6b, the effect of wind speed is primarily manifested by modulating the molecular propagation speed, which reduces the time required for BVOCs to reach the receiving plants. However, since the distance remains the same, the maximum BVOC absorption amount remains unchanged. Higher wind speeds enable plants to acquire sufficient BVOCs in less time, thereby facilitating the more rapid recognition of environmental stress signals and triggering timely stress responses.

### 3.2. Impact of Factors on Demodulation Performance

To date, over 1400 distinct stress types have been documented in plants, among which this study specifically focuses on pest stress and heat stress. Under pest stress conditions, the release of methyl jasmonate (MeJA) by plants has been identified as a key mediator of long-distance inter-plant stress signaling [33]. In contrast, ethanol (C_2_H_5_OH) released under heat stress is closely associated with the severity of plant heat stress [34]. This section will use these two BVOCs as subjects for further experiments, examining how factors such as thresholds and signal-to-noise ratio (SNR) affect the system’s DER. For clarity, this paper will represent MeJA as $b_{1}$ and C_2_H_5_OH as $b_{2}$.

#### 3.2.1. Impact of Threshold

**Figure 7:**
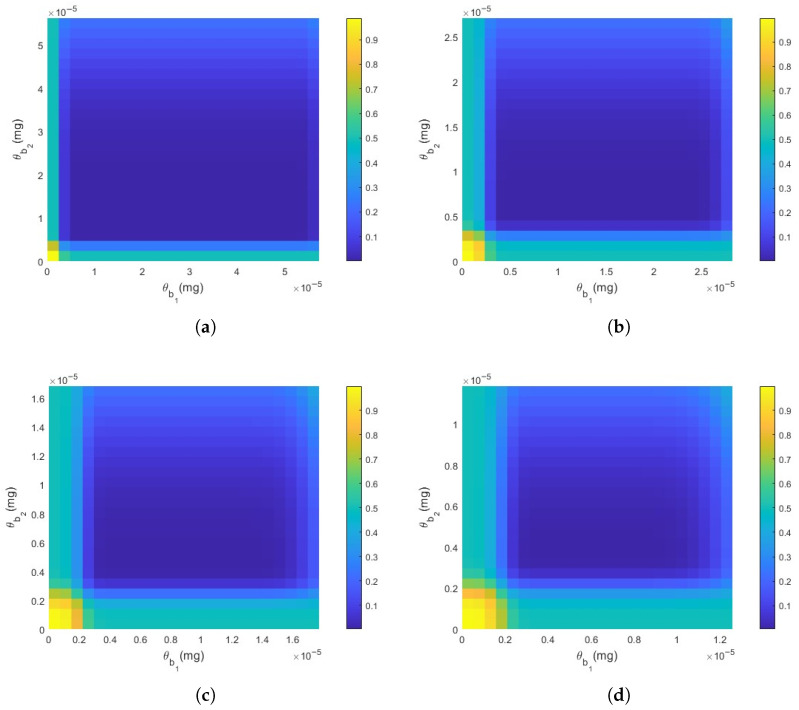
The impact of thresholds on DER. (**a**) M=0.0016mg, d=1m, v=1m/s, $D_{b1}=0.007\;m^{2}/s$, $D_{b2}=0.009\;m^{2}/s$, τ=2s, ω=2s, ϕ=0.5, SNR=10. (**b**) d=2m. (**c**) d=3m. (**d**) d=4m. All other parameters remain constant.

In this section, we analyze the relationship between the different thresholds and the DER. As shown in Figure 7, the DER first decreases and subsequently increases with the elevation of both threshold values. Specifically, the deeper blue region represents a lower DER, indicating that the system performs more effectively at certain optimal threshold values. A further comparison of Figure 7a,d reveals that as the distance increases, the area of the blue region indicating optimal performance exhibits a decreasing trend, indicating that distance has a negative impact on detection performance. Therefore, selecting appropriate threshold values based on distance is crucial for maintaining optimal detection and response performance.

#### 3.2.2. Impact of SNR

**Figure 8:**
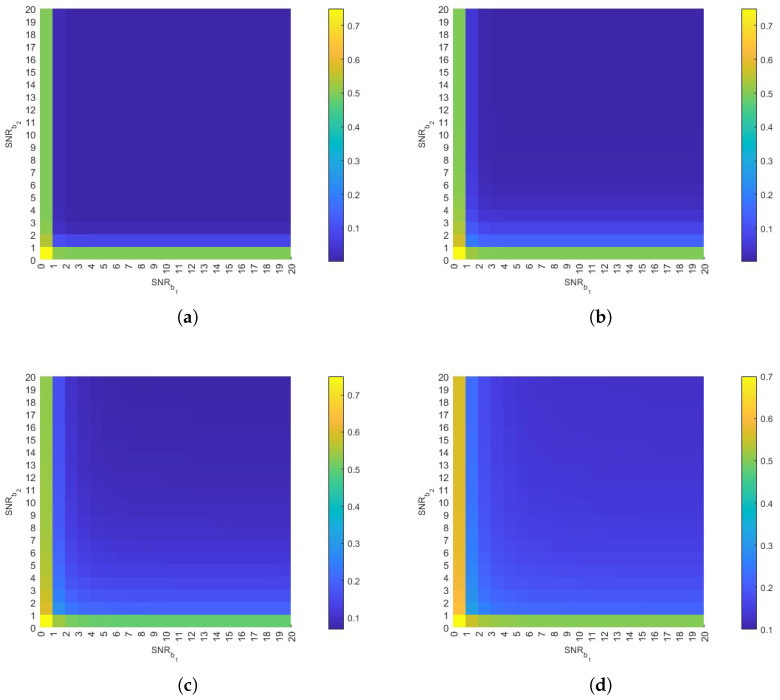
The impact of SNR on DER. (**a**) M=0.0016mg, d=1m, v=1m/s, $D_{b1}=0.007\;m^{2}/s$, $D_{b2}=0.009\;m^{2}/s$, τ=2s, ω=2s, ϕ=0.5. (**b**) d=2m. (**c**) d=3m. (**d**) d=4m. All other parameters remain constant.

This section examines the relationship between the SNR of both $b_{1}$ and $b_{2}$ and the DER. The SNR is defined as the ratio of signal power to noise power [35]. As shown in Figure 8, the DER decreases with increasing SNR. This inverse relationship occurs because higher SNR values indicate reduced interference from neighboring plants’ molecules, thereby mitigating their impact on BVOCs during the current time slot. Furthermore, as demonstrated in Figure 8d, the DER increases more significantly under low SNR conditions when the distance increases. This occurs because the increased distance leads to a reduction in the amount of BVOCs reaching the receiver, resulting in a higher proportion of interference molecules from other plants. This behavior underscores the dual dependence of DER on both distance and SNR. Therefore, system design ought to incorporate adaptive thresholding to enhance robustness under conditions of low SNR and long transmission distances. Crucially, the results show that even at increased distances, a high SNR can maintain the DER at a relatively low level. This confirms that a high SNR effectively counteracts performance degradation caused by both distance and interference, highlighting its critical role in ensuring reliable communication performance.

#### 3.2.3. Impact of Transmitted Quality

**Figure 9:**
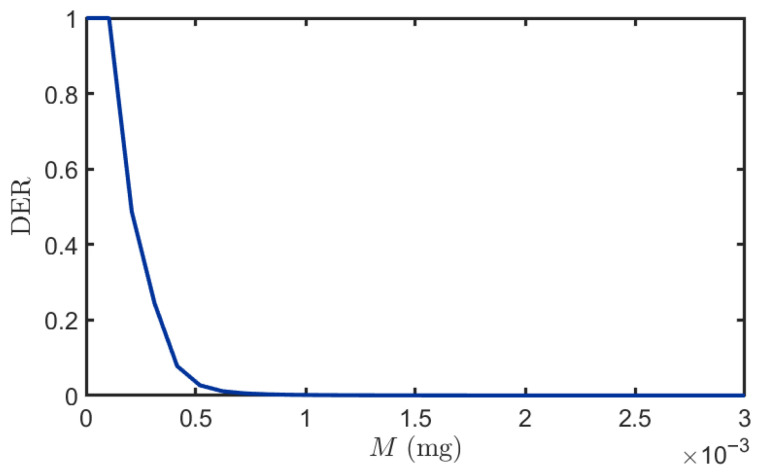
The impact of different emission levels on DER. v=1m/s, $D_{b1}=0.007\;m^{2}/s$, $D_{b2}=0.009\;m^{2}/s$, τ=2s, ϕ=0.5.

Figure 9 demonstrates an inverse relationship between DER and the quantity of BVOCs released by the transmitter, reflecting improved system performance with increased emission. At low emission levels *M* (0 mg to 0.00003 mg), DER reaches 1 due to either complete BVOC depletion in the channel preventing molecular arrival at the receiver, or due to interference from neighboring plant molecules causing signal detection failure. As *M* increases, the DER decreases progressively due to enhanced BVOC flux reaching the receiver, enabling sufficient molecular absorption by leaves to exceed the minimum detection threshold. Furthermore, the steep initial decline indicates high sensitivity to emission changes in the low-power regime, while the eventual saturation above approximately M (0.001 mg) suggests the existence of a fundamental performance bound. This limitation may arise from either the maximum capacity of stomatal absorption rate, enzymatic reaction velocity, or intracellular signal transduction pathways in the receiver plant leaves, or from the inherent maximum mutual information rate of the molecular diffusion channel itself.

### 3.3. An MC Testbed

To accurately simulate the overall process of plant stress detection, we established an MC test bed. This platform provides a controlled experimental environment capable of simulating the release, propagation, absorption, and detection of BVOCs from plants under stress conditions, thereby validating the proposed theoretical model.

#### 3.3.1. Basic Parts of the Testbed

In this section, the platform we constructed is an extension and improvement of the MC testbed presented in [36], aiming to reproduce the overall process by which plants detect stress through the recognition of BVOCs in natural environments.

**Figure 10:**
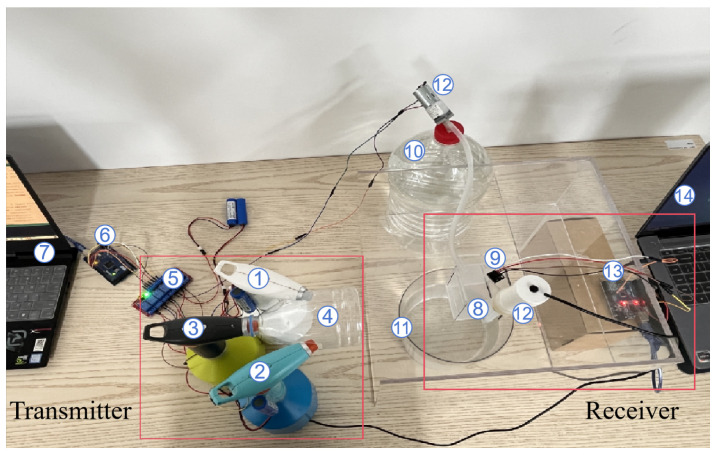
MC testbed.

Figure 10 illustrates the overall structure of the platform. The transmitter module primarily consists of three electronic aerosolizers and a collection pipe. The white aerosolizer (Aerosolizer 1, PH-1001, Xie Bingran Co., Ltd., Ningbo, China) contains red pigment dye, representing the BVOCs emitted by plants under a specific abiotic stress; the blue aerosolizer (Aerosolizer 2, PH-1001, Xie Bingran Co., Ltd., Ningbo, China) is filled with blue pigment dye, symbolizing the BVOCs released by plants under a particular biotic stress. The black aerosolizer (Aerosolizer 3, PH-1001, Xie Bingran Co., Ltd., Ningbo, China) is used to periodically clean any residual pigment molecules in the air after each emission, thus minimizing the impact of previously emitted pigment on the experiment and ensuring the consistency of each spray event. The collection device (Device 4) connects the three aerosolizers and is responsible for gathering the released pigment molecules, ensuring that the pigment entering the air channel originates from the same position. Additionally, the transmitter is equipped with a control system, which includes a relay (Relay 5, Risym 12V, Shenzhen Kobe Electronics Technology Co., Ltd., Shenzhen, China) and an Arduino development board (Board 6, Arduino UNO, Arduino AG, Turin, Italy). The relay connects the development board to the electronic aerosolizers via jumper wires, enabling effective signal transmission and precise control. The other end of the Arduino board is connected to a laptop (Laptop 7), where a program runs to precisely control the emission behavior of the three electronic aerosolizers. We can set the working parameters of the aerosolizers through the computer interface, such as spraying frequency, duration, and amount, in order to simulate a variety of environmental scenarios.

The structure of the receiver is relatively complex, comprising an open container (Container 8), a spectral sensor (Sensor 9, As7341, Longkexin Technology Co., Ltd., Jinhua, China), a water reservoir (Reservoir 10), a waste recovery container (Container 11), two small pumps (Pump 12), another Arduino development board (Board 13, Arduino UNO, Arduino AG, Turin, Italy), and a laptop (Laptop 14). Before the experiment starts, 20 mL of water is first injected into the open container as a prerequisite. When the pigment molecules emitted by the electronic aerosolizers reach the open container, they dissolve in the water to form a colored solution, thus simulating the process by which plant leaves absorb BVOCs. As the color of the solution gradually changes, the spectral sensor continuously collects the light intensity data of the colored solution and transmits these data to the laptop via the Arduino development board—this enables visual data analysis to identify which type of pigment molecule was released by the transmitter module. After the spectral sensor completes the detection, both pumps commence operation. One pump draws the colored solution from the open container into the waste recovery container to remove any residual liquid and prevent the pigment molecules from affecting subsequent experiments; the other pump injects pure water into the open container, preparing it for the next reception.

#### 3.3.2. Experimental Results

In this section, we utilized the testbed to simulate the entire process of stress detection. To ensure the validity of the experiment, we employed red and blue pigments to represent pest stress and high-temperature stress, respectively, with the red pigment molecule denoted as $b_{1}$ and the blue pigment molecule denoted as $b_{2}$. Prior to the experiment, these pigment molecules were pre-stored in electronic Aerosolizer 1 and Aerosolizer 2. In the experimental setup, the sending program encodes $b_{1}$ as 0 and $b_{2}$ as 1. When the program input is 0, Aerosolizer 1 is activated to release a specific volume of pigment molecules; when the input is 1, Aerosolizer 2 is activated and releases the same volume of pigment molecules. During each time slot, only one type of pigment molecule is allowed to be released—this ensures the clarity and accuracy of experimental data. The released molecules propagate through the air channel, ultimately reaching the receiver. At the receiver module, the spectral sensor is used to read the light intensity values of the red or blue solution in Container 8. This measurement enables the differentiation of whether red or blue pigment was released in the current time slot, and thus allows for the determination of the type of source stress input at the transmitter end.

**Figure 11:**
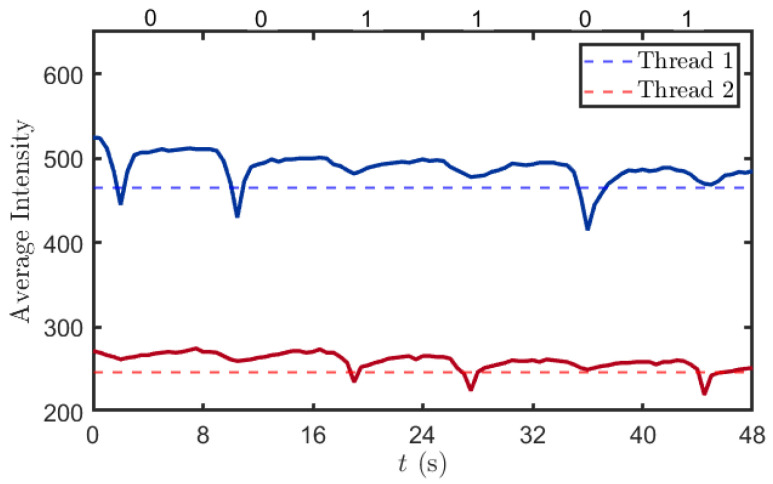
Detection results. The red and blue curves represent the variation in parameters of the optical transmission sensor, where the red dashed line denotes the value of parameter $b_{1}$ and the blue dashed line denotes the value of parameter $b_{2}$. During each time interval, if there is only one curve with the lowest point corresponding to the dashed line, the respective parameter would be successfully demodulated.

In the experiment, we defined the transmission sequence at the transmitter module as “001101.” As shown in Figure 11, if red pigment is transmitted in the current time slot, the blue channel values of the spectral sensor exhibit a significant decrease. This is because red pigment has a pronounced absorption peak in the blue–green wavelength range (450–500 nm). Since red and blue are complementary colors, an increase in the concentration of red pigment in the solution enhances its absorption of blue light, leading to a reduction in the light intensity value detected by the blue channel of the spectral sensor. Similarly, if blue pigment is transmitted in the current time slot, the corresponding value in the red channel of the spectral sensor will also show a significant decrease. Based on the above-described principles, we established two thresholds (465 and 247); values below these thresholds indicate the successful detection of the target pigment, thereby enabling the identification of the pigment molecules in the current time slot.

In the first 8 s, the blue channel reading decreased from 525 to 458 before rising back to approximately 510. This fluctuation is attributed to the pump-driven removal of the solution from the receiving container, followed by the injection of clear water. The introduction of clear water led to a recovery of the light intensity in the blue channel to a higher value, although it did not return completely to the initial level. This is due to the residual solution extracted by the pump, which left some pigment molecules still retained in the container. In contrast, the variation in the red channel readings was relatively minimal, primarily because the red pigment solution exhibits strong absorption of blue light while its absorption of red light is comparatively weak.

## 4. Discussion

This study investigates the transmission of stress signals between plants via BVOCs and establishes a comprehensive theoretical model that covers the entire process of BVOC generation, propagation, and detection. Unlike conventional symptom-based or imaging-based techniques, our approach leverages MC principles to differentiate stress types by modulating distinct stress signals into corresponding BVOC profiles. Specifically, a stressed transmitter plant releases specific BVOCs, which are then demodulated by the receiver based on molecular identity and absorption quantity according to predefined decision rules, enabling chemistry-based discrimination between biotic and abiotic stresses. Furthermore, we analyzed how key factors such as transmission distance, wind speed, and emission quantity affect BVOC absorption. We also formulated the DER to quantitatively evaluate system performance in discriminating pest and heat stress.

To accurately simulate the overall process of plant stress detection and experimentally validate the proposed MC framework, we established an MC testbed. This platform provides a controlled experimental environment specifically designed to physically instantiate key components of the theoretical model—aerosolizers simulate the transmitter releasing stress-specific molecules, the air channel represents the propagation environment, and the spectral sensor acts as the receiver detecting and demodulating the molecular signals. By aligning each experimental component with its theoretical counterpart, the testbed enables the validation of the entire signal chain—from modulated release to received concentration-based stress identification—under adjustable and repeatable conditions.

When compared to existing platforms, such as systems using live Mimosa pudica plants to transmit information via calcium ion flows and electrical signals [37] or synthetic agrotechnology testbeds designed for detecting volatile signals related to fruit rot [38], our setup employs pigment proxies rather than complex biological or chemical agents. This design choice simplifies implementation and enhances interpretability. However, this streamlined approach also reduces biological fidelity and environmental complexity. Despite these trade-offs, our testbed advances current methodologies by explicitly incorporating dynamic environmental modulation and stress-specific molecular modulation, thus providing an accessible and conceptually clear platform for the initial validation of interspecific plant communication mechanisms under controlled conditions.

However, when compared to actual natural environments, the current model still presents several limitations. Firstly, it does not fully address the complex chemical signaling systems arising from the stress-induced synergistic release of multiple BVOCs. Secondly, environmental variables including temperature, humidity, wind speed, and topography collectively influence molecular propagation behavior and reception probabilities in ways that are not currently captured by the model. Moreover, important physical properties of BVOCs such as gravitational effects and evaporation processes remain unincorporated. For instance, gravity affects BVOC dispersion by causing the rapid settling of heavier molecules, particularly restricting transmission range between tall plant species. Under high-temperature and low-humidity conditions, accelerated evaporation rates diminish BVOC concentrations near receiver plants, directly reducing detection sensitivity. Additionally, the channel model presumes an infinite environment in all directions, which considerably simplifies the problem. In reality, even in straightforward agricultural settings, the ground acts as an absorbing boundary condition, which alters molecular diffusion patterns. Finally, the current testbed has only preliminarily validated the model’s rationality and considered only the detection processes of two types of stress.

Future research will focus on overcoming the current model’s limitations to enhance its applicability and accuracy in real-world environments. We plan to incorporate additional environmental variables, such as temperature, humidity, and wind speed, to construct a more complex multivariable model, enabling a quantitative analysis of the combined effects of these factors on molecular propagation dynamics. Notably, the ground boundary effect will be explicitly integrated by implementing an absorptive boundary condition at the soil–air interface, thereby allowing a more realistic simulation of semi-bounded agricultural environments. Furthermore, we will continue to improve the testbed to simulate a wider variety of stress detection processes, thereby providing more precise support for the development of plant stress information transmission mechanisms.

## Figures and Tables

**Table 1:** Parameters settings.

Parameter	Value
Leaf area ($A_{L}$)	$0.009\;m^{2}$
Leaf electric conductance (*g*)	86.4m/day
Leaf density ($\rho_{L}$)	$1000\;{\mathrm{kg}/m}^{2}$
Leaf quality ($m_{L}$)	0.05kg
Leaf–air partition coefficient ($K_{L}$)	20
Sphere radius (*R*)	0.15m

## Data Availability

Data are contained within the article.
